# Tyrosine kinase-mediated axial motility of basal cells revealed by intravital imaging

**DOI:** 10.1038/ncomms10666

**Published:** 2016-02-12

**Authors:** Jeremy Roy, Bongki Kim, Eric Hill, Pablo Visconti, Dario Krapf, Claudio Vinegoni, Ralph Weissleder, Dennis Brown, Sylvie Breton

**Affiliations:** 1 Program in Membrane Biology, Nephrology Division and Center for Systems Biology, Massachusetts General Hospital/Harvard Medical School, Boston, Massachusetts 02114, USA; 2 Department of Veterinary and Animal Science, University of Massachusetts, Amherst, Massachusetts 01003, USA

## Abstract

Epithelial cells are generally considered to be static relative to their neighbours. Basal cells in pseudostratified epithelia display a single long cytoplasmic process that can cross the tight junction barrier to reach the lumen. Using *in vivo* microscopy to visualize the epididymis, a model system for the study of pseudostratified epithelia, we report here the surprising discovery that these basal cell projections—which we call axiopodia—periodically extend and retract over time. We found that axiopodia extensions and retractions follow an oscillatory pattern. This movement, which we refer to as periodic axial motility (PAM), is controlled by c-Src and MEK1/2–ERK1/2. Therapeutic inhibition of tyrosine kinase activity induces a retraction of these projections. Such unexpected cell motility may reflect a novel mechanism by which specialized epithelial cells sample the luminal environment.

## Abstract (web summary)

It was recently shown that basal cells in pseudostratified epithelia extend a long cytoplasmic process across the tight junction barrier into the lumen. Here Roy & Kim *et al*. show that these projections, which they call axiopodia, extend and retract over time in a c-Src and MEK-ERK-dependent manner.

## Graphical abstract

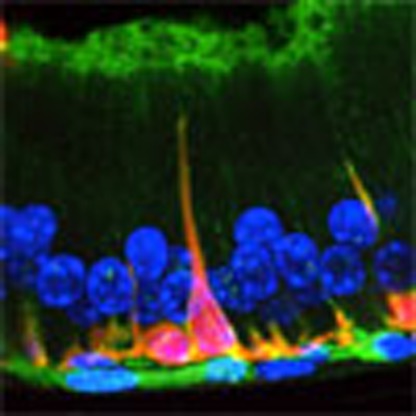

Crosstalk between different cell types is crucial for the dynamic function of epithelia. We previously reported that basal cells (BCs) in pseudostratified epithelia extend an intraepithelial narrow body projection that can cross the tight junction barrier to survey the luminal environment. In the rat epididymis, BCs detect luminal angiotensin II, and they then communicate their finding to adjacent epithelial cells to increase luminal acidification, a process that is crucial for proper sperm maturation and storage^1^. This revealed that BCs are both sensors of luminal stimuli and transmitters that modulate epithelial function via crosstalk with neighbouring cells. Interestingly, a heterogeneous population of BCs with intercellular projections of various lengths was detected in all pseudostratified epithelia examined^1^,^2^,^3^,^4^,^5^,^6^. This raises the question whether BCs are dynamic cells that can regulate the length of their projections, or whether there are distinct subsets of BCs, some with luminal-sensing properties and some without. To distinguish between these possibilities, we developed transgenic mice in which the red fluorescent protein, tandem dimer (td) Tomato (tdTomato)^7^, is expressed under the control of the promoter of keratin 5 (KRT5), a specific marker of BCs. These mice were used in combination with two-photon fluorescence intravital microscopy (FIVM) to characterize the temporal and spatial characteristics of individual BCs visualized in real time in the epididymis of living animals^8^,^9^,^10^,^11^. We show that BCs have a dynamic structural plasticity: they continuously extend and retract their intercellular projections—here referred to as axiopodia—along their basal-to-apical axis. We found that the axiopodium length follows an oscillatory pattern of extension/retraction, a process that is repressed after therapeutic inhibition of tyrosine kinases, and is regulated by the c-Src and MEK1/2/ERK1/2 pathways. We name this dynamic movement of axiopodia periodic axial motility (PAM). Inhibition of PAM may contribute to the adverse effect of tyrosine kinase inhibitors used for cancer treatment on male fertility^12^.

## Results

### Generation and characterization of transgenic reporter mice

**Figure 1:**
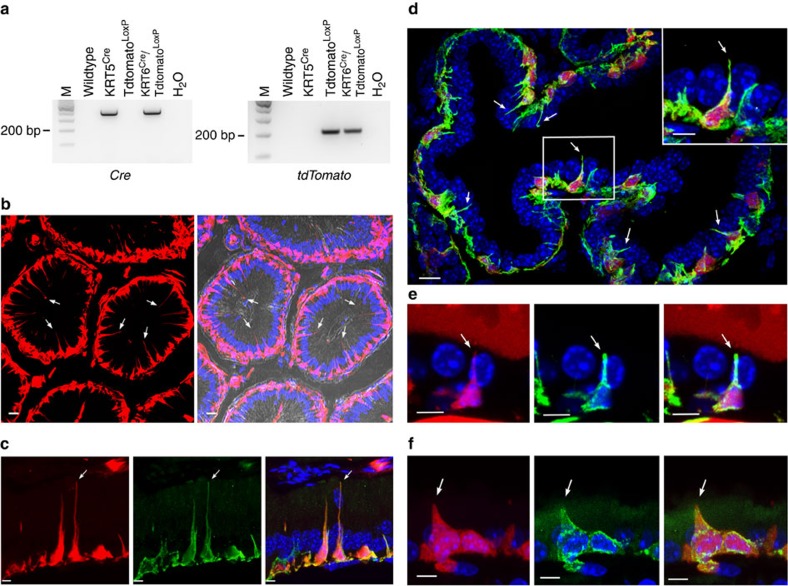
Characterization of KRT5^Cre^/tdTomato^LoxP^ mice. (**a**) PCR detection of Cre recombinase and tdTomato from genomic DNA. The Cre recombinase gene was detected in KRT5^Cre^ and KRT5^Cre^/tdTomato^LoxP^ mice, but not in WT and tdTomato^LoxP^ mice (left). The *tdTomato* gene was detected in DNA isolated from tdTomato^LoxP^ and KRT5^Cre^/tdTomato^LoxP^ mice, but not from WT and KRT5^Cre^ mice (right). No products were detected in the H_2_O lanes. (**b**) Proximal epididymis visualized by epifluorescence microscopy (left), and together with differential interference contrast (right). Strong tdTomato fluorescence (red) was detected in a subset of epithelial cells, identified as BCs by the location of their nuclei (blue) in the basal region of the epithelium. Several BCs with long intercellular cell body projections (axiopodia) were detected (arrows). Double-labelling for KRT5 (green) confirmed expression of tdTomato in BCs in the proximal epididymis ((**c**) see also immunofluorescence examination of the proximal epididymis (IS; Fig. 1b,c and Supplementary Movie 1)), prostate (**d**), seminal vesicles (**e**) and trachea (**f**). In all these pseudostratified epithelia, a subset of BCs have an axiopodium extending in the direction of the lumen (arrows). Nuclei are labelled in blue with 4,6-diamidino-2-phenylindole (DAPI). Scale bars (**b**,**d**), 10 μm; (**c**,**d** (inset), **e**,**f**), 5 μm.

We generated a novel transgenic mouse that expresses tdTomato under the control of the promoter of the BC-specific gene *KRT5*. PCR analysis shows expression of Cre recombinase in KRT5^Cre^ and KRT5^Cre^/tdTomato^LoxP^ mice (Fig. 1a, left), and expression of the *tdTomato* gene in tdTomato^LoxP^ and KRT5^Cre^/tdTomato^LoxP^ mice (Fig. 1a, right). These results indicate the expression of tdTomato in KRT5-expressing cells in the KRT5^Cre^/tdTomato^LoxP^ mice. This is confirmed with immunofluorescence examination of the proximal epididymis (initial segment: IS; Fig. 1b,c, Supplementary Movie 1), prostate (Fig. 1d), seminal vesicles (Fig. 1e) and trachea (Fig. 1f). Double immunolabelling for KRT5 (green) confirms expression of tdTomato in BCs. Several tdTomato-positive BCs have an intercellular projection extending in the direction of the lumen (axiopodia; arrows). No tdTomato fluorescence is detected in the efferent ducts, which do not contain BCs. These results indicate that the presence of axiopodia is a common feature of BCs in pseudostratified epithelia.

### Three-dimensional imaging of BCs using confocal microscopy

**Figure 2:**
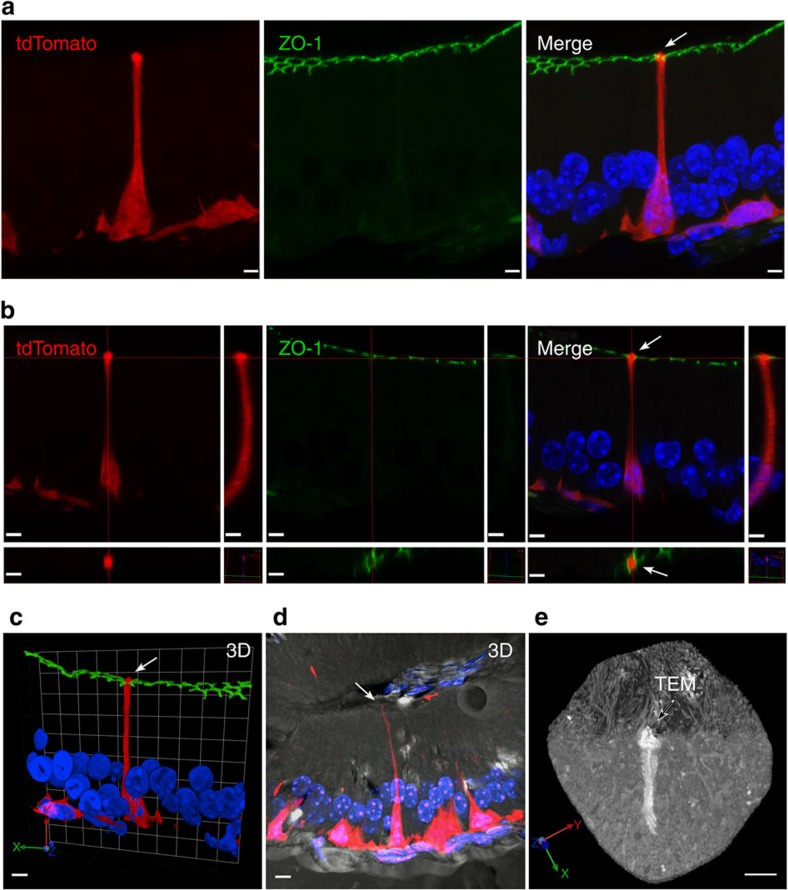
Basal cell axiopodia can cross the tight junction barrier. (**a**–**c**) Laser scanning confocal microscope images of a 16-μm cryosection of the proximal epididymis of a KRT5^Cre^/tdTomato^LoxP^ mouse labelled for the tight junction marker ZO-1 (green), showing an extended focus view (**a**), slice view (**b**) and a still image of a 3D reconstruction of the BC (**c**; see immunofluorescence examination of the proximal epididymis (IS; Fig. 1b,c and Supplementary Movie 2)). All images show that the axiopodium of the tdTomato-positive BC (red; arrows) is in contact with the luminal side of the epithelium, delineated by the ZO-1-labelled tight junctions. (**d**) 3D reconstruction of another tdTomato-positive BC, superimposed on a differential interference contrast image, showing that its axiopodium is in contact with the luminal compartment (arrow; see also immunofluorescence examination of the proximal epididymis (IS; Fig. 1b,c and Supplementary Movie 3). Nuclei and sperm are labelled in blue with DAPI. (**e**) Epididymis was immunostained using an antibody that recognizes tdTomato, and a stack of ultrathin serial sections were obtained using electron microscopy. Images from these serial sections were superimposed using Fiji and imported into the Volocity software to generate a ‘3D' projection view of the cells. The ‘apical' pole of this BC is in contact with the luminal compartment and shows the presence of numerous short TEM (arrow, see also immunofluorescence examination of the proximal epididymis (IS; Fig. 1b,c and Supplementary Movie 4). Longer stereocilia emanating from adjacent principal cells are also seen. Scale bars (**a**–**d**), 5 μm; (**e**), 2 μm.

We then examined tdTomato-positive BCs in the epididymis using confocal laser scanning microscopy and electron microscopy. Different confocal imaging views of the same cell (Fig. 2a–c, see also immunofluorescence examination of the proximal epididymis (IS; Fig. 1b,c and Supplementary Movie 2)) clearly demonstrate that the axiopodium of the BC (red) reaches the luminal compartment by crossing the tight junction barrier, labelled for ZO-1 (green). Figure 2d shows a three-dimensional (3D) reconstruction of another tdTomato-positive BC superimposed with a bright-field differential interference contrast image, also showing that its axiopodium is in contact with the luminal side of the epithelium (see also immunofluorescence examination of the proximal epididymis (IS; Fig. 1b,c and Supplementary Movie 3). This is confirmed using electron microscopy of a BC, after pre-embedding labelling with an antibody against tdTomato (Fig. 2e; see also immunofluorescence examination of the proximal epididymis (IS; Fig. 1b,c and Supplementary Movie 4). In this image, the apical region of a BC is visualized in serial ultrathin sections, which were then superimposed to generate a *3D* projection view of the cell. This high-resolution image shows the presence of small transepithelial microvillar (TEM) projections (arrow) emanating from the portion of the cell that is in contact with the luminal compartment.

### Four-dimensional imaging of BCs using FIVM

**Figure 3:**
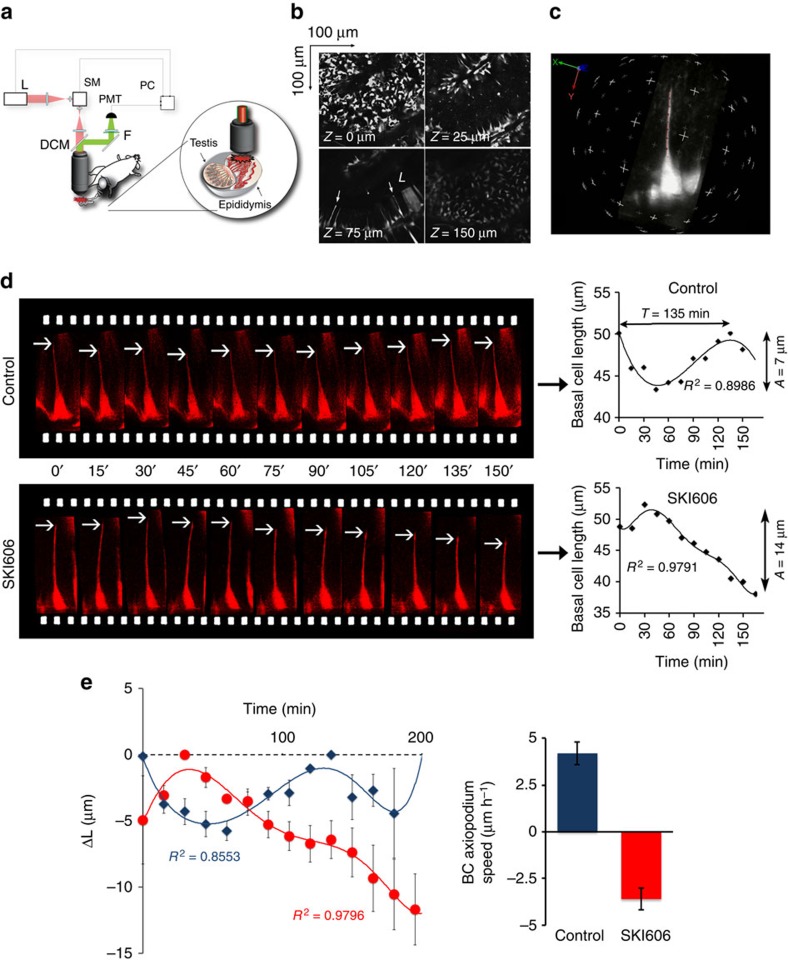
Periodic axial motility of BC axiopodia visualized by two-photon FIVM. (**a**) Excitation light (red beam) from a Ti:sapphire laser (L) is directed into a laser scanning microscope (SM) and focused onto the exposed epididymis. Emitted fluorescence (green beam) is epi-collected after reflection from a dichroic mirror (DCM), and filtered with a bandpass filter (F) before non-descanned detection (PMT). Acquisition, automatic positioning and axial coordinates are controlled by a computer (PC). Inset: The exposed epididymis was immersed in a thermal bath and a water immersion objective was positioned over the surface of the IS. (**b**) A stack of optical images was acquired along the *z* axis through the thickness of the IS tubule (see also immunofluorescence examination of the proximal epididymis (IS; Fig. 1b,c, Supplementary Movie 5 and Supplementary Fig. 1). In the bottom left panel, arrows show BC axiopodia that extend towards the lumen (*L*). (**c**) 3D reconstruction of a single BC from a higher-magnification Z-stack series (see also immunofluorescence examination of the proximal epididymis (IS; Fig. 1b,c and Supplementary Movie 6). (**d**) Top left panel: time-lapse images of an individual live BC from a control mouse. The arrows show the tip of the axiopodium visualized at 15-min intervals. The length of this BC, taken from the base of the cell to the tip of the axiopodium, was plotted over time (top, right panel). The line shows the best-fit curve for this data set. Bottom, left panel: time-lapse images of an individual live BC from a SKI606-treated mouse. The length of this BC was plotted over time (bottom, right panel). The line shows the best-fit curve for this data set. (**e**) Left: average BC length (*L*) plotted versus time. Individual BC data sets were synchronized in time according to the maximum length *LMj* measured for each cell Cj. The difference in length from this maximum value (Δ*L*(*t*)=*Lj*(*t*)−*LMj*) was calculated for each cell Cj and the average values (mean±s.e.m.) were plotted versus time (control: blue lozenges, SKI606-treated: red circles). The lines show the best-fit curve for each data set (control: blue line, SKI-treated: red line). Right: variation of BC length versus time: Δμm h^−1^ for control and SKI606-treated animals. The positive value indicates elongation and the negative value indicates retraction of BC axiopodia.

We tested here the hypothesis that BCs are dynamic cells that undergo morphological plasticity by imaging the epididymis of KRT5^Cre^/tdTomato^LoxP^ mice in real time and *in vivo* using two-photon FIVM^8^,^9^,^10^,^11^. The epididymis is an ideal organ for FIVM: located in the scrotal sac, it is easily externalized and does not require post-acquisition motion artefact correction^11^. We developed a minimally invasive surgical procedure to expose the epididymis while maintaining the blood flow and connection to the testis intact (Fig. 3a). The IS was first visualized at low magnification (Fig. 3b; see also immunofluorescence examination of the proximal epididymis (IS; Fig. 1b,c, Supplementary Movie 5 and Supplementary Fig. 1)). Higher-magnification 3D reconstructions of single BCs, identified by their red fluorescence, were then generated (Fig. 3c; see also immunofluorescence examination of the proximal epididymis (IS; Fig. 1b,c and Supplementary Movie 6)). Figure 3d (top panel) shows a filmstrip of the projected view of a single representative BC imaged every 15 min for 150 min from a control mouse. Arrows denote the tip of the BC axiopodium. The length of this BC is plotted over time in Fig. 3d (top right panel). A curve fitting analysis (black line) shows an oscillatory pattern of retraction followed by extension of the BC axiopodium, with an amplitude (maximum length−minimum length) of 7 μm—representing 17% of the axiopodium maximum length—and a period of *T*=135 min. Examination of several BCs in different mice showed a similar oscillatory movement pattern. Each individual BC has its own phase of oscillation. To allow for a systematic comparison of their retraction and elongation, all individual BC data sets were synchronized in time (Fig. 3e). A curve fitting analysis shows that, on average, BC axiopodia exhibit an oscillatory pattern of retraction and extension, with an amplitude of 5 μm in control mice (blue line: *n*=9 BCs from six mice, *R*^2^=0.8553). We term this movement pattern PAM.

Tyrosine kinases play a crucial role in male fertility and are major regulators of epithelial cell differentiation in the male reproductive tract^13^,^14^,^15^,^16^,^17^. Importantly, tyrosine kinase inhibitors, including SKI606 (also known as Bosulif or Bosutinib), were recently approved for the treatment of some cancers^18^. These drugs were suggested to have adverse effects on male fertility^12^, and we tested here the effect of SKI606 on axiopodia plasticity. To do so, SKI606 (30 mg kg^−1^) was injected through the tail vein while BCs were examined every 15 min using FIVM. As shown in Fig. 3d, after an initial elongation of the BC axiopodium (bottom panels), a retraction is initiated 30 min after injection, and the axiopodium becomes 14 μm smaller after 3 h. Visualization of several BCs under these conditions confirmed this pattern (Fig. 3e, red circles and red line, *n*=7 BCs from three mice). On average, BCs start near their maximum length and their axiopodia become 12±2 μm (mean±s.e.m.) shorter over a 3-h period in the presence of SKI606. In control mice, the velocity of movement at which BC axiopodia extend, calculated from the slope between the minimum and maximum BC lengths, is 4.2±0.6 μm h^−1^, whereas in SKI606-treated mice the speed is reversed in direction to −3.6±0.6 μm h^−1^ (Fig. 3e, right panel).

We further explored the inhibitory effect of SKI606 on PAM by examining a larger population of BCs with conventional fluorescence microscopy. After FIVM imaging, each epididymis was fixed and cryosections were made. BCs were examined and distributed into different categories depending on the length of their axiopodia. SKI606 induces a significant reduction in the percentage of BCs with long axiopodia that reached the apical border of the epithelium (9.3±0.5% versus 18.9±2.5%, *n*=3 SKI606-treated versus *n*=7 control mice; *P*<0.05 by analysis of variance (ANOVA) followed by Student's *t*-test) and a significant increase in the number of BCs with no axiopodia (22.1%±1.3% versus 17.8±2.0%; *P*<0.05 by ANOVA followed by Student's *t*-test). These results further demonstrate the retraction of BC axiopodia induced by SKI606.

### The c-Src signalling pathway regulates PAM

**Figure 4:**
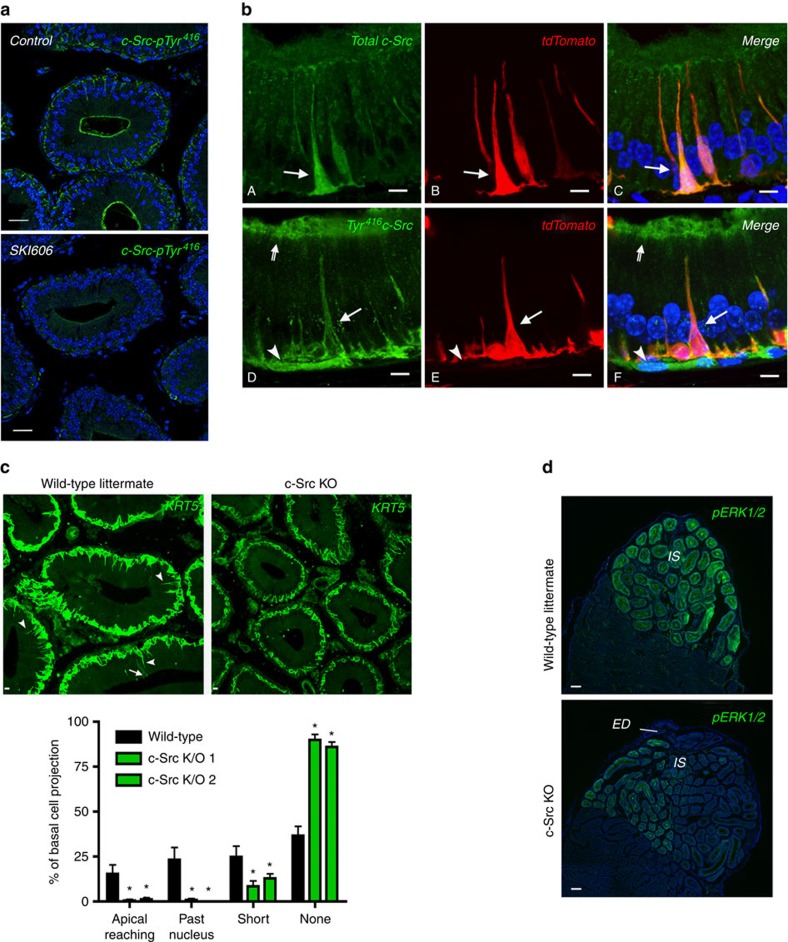
Role of c-Src in the formation of BC axiopodia. (**a**) SKI606 induced a significant reduction in c-Src Tyr^416^ phosphorylation (green; bottom) compared with control (top). Scale bars, 10 μm. (**b**; A–C) Immunofluorescence labelling using an antibody that recognizes total c-Src (A: green, C: orange) showed its high expression in tdTomato-positive BCs (B: red, C: orange), where it is distributed throughout the cytoplasm (arrows). A weaker c-Src labelling was also detected in adjacent principal cells. (D–F) Phosphorylated c-Src at residue Tyr^416^ (D: green, F: orange) is enriched near the plasma membrane (arrows) of tdTomato-positive BCs (E: red, F: orange), and in the apical stereocilia of principal cells (double-tailed arrows). Positive c-Src-pTyr^416^ labelling was also detected in a few cells located below BCs (arrowheads). Scale bars, 5 μm. All images represent extended focus views from a series of confocal images that were acquired from 16-μm tissue sections. (**c**) Cryostat sections (5 μm) of the IS of WT (left) and c-Src KO (right) littermates were labelled for KRT5 (green) to visualize BCs. The arrow in A indicates a BC axiopodium that reaches the apical border of the epithelium (apical reaching), and the arrowheads show BCs with intermediate length axiopodia (past nucleus). No BCs with long or intermediate axiopodia were detected in c-Src KO mice. Scale bars, 5 μm. (Bottom panel) Quantification showed no BCs with medium length (past nucleus) and long (apical reaching) axiopodia in c-Src KO mice. *P<0.05 compared with WT by ANOVA followed by Student's *t*-test. (**d**) Mosaic (× 20) images of the proximal epididymis of WT and c-Src knockout littermates immunolabelled for the pERK1/2. pERK1/2 labelling was weaker in the IS of c-Src KO mice compared with control. ED: efferent ducts. Scale bars, 50 μm. (**a**,**b**,**d**) Nuclei were labelled in blue with DAPI.

SKI606 inhibits a variety of tyrosine kinases including the non-receptor tyrosine kinase cellular-sarcoma c-Src^18^. We tested the effect of SKI606 on c-Src phosphorylation using an antibody that recognizes c-Src when phosphorylated at residue Tyr^416^ (the active form of c-Src). We found a significant reduction in c-Src Tyr^416^ phosphorylation (c-Src-pTyr^416^) in the IS of SKI606-treated mice compared with controls (Fig. 4a), showing efficacy of the treatment and confirming c-Src kinase inhibition by SKI606. The specificity of this antibody for c-Src-pTyr^416^ has been shown previously in capacitated spermatozoa^19^. In addition, its specificity was further demonstrated by the absence of labelling in epididymal BCs in c-Src knockout (KO) mice compared with WT mice (Supplementary Fig. 2).

Higher-magnification images of *BC-tdTomato* mouse epididymis using an antibody that recognizes total c-Src showed enrichment of the protein in tdTomato-positive BCs (arrows) compared with adjacent epithelial cells (Fig. 4bA–C). c-Src is distributed throughout the cytoplasm and nucleus of BCs. A weaker c-Src signal is also detected in adjacent principal cells. Phosphorylated c-Src at the Tyr^416^ residue is detected along the membrane of tdTomato-positive BCs (Fig. 4bD–F; arrows), as well as in the apical stereocilia of principal cells (double-tailed arrows). Some cells located below tdTomato-positive BCs are also positive for c-Src-pTyr^416^ (arrowheads). The participation of c-Src in the regulation of BC axiopodia PAM was examined using c-Src KO mice. The epididymides of c-Src KO and their wild-type (WT) littermates were labelled for KRT5, and the morphological characteristics of BCs were examined (Fig. 4c). Very few BCs with an axiopodium are observed in the c-Src KO mice compared with WT (top panels). Quantification shows a significant decrease in the number of BCs with (1) long axiopodia that reached the apical border of the epithelium (top panels: arrow, bottom panel: apical reaching); (2) axiopodia that reached past the rows formed by the nuclei in adjacent principal cells (top panels: arrowheads, bottom panel: past nucleus); and (3) short axiopodia (bottom panel: short) in c-Src KO mice compared with WTs. A concomitant increase in the number of BCs with no axiopodia (bottom panel: none) is observed in c-Src KO mice. There is no statistically significant difference in the total number of BCs per tubule cross-sections.

### MEK1/2/ERK1/2 signalling regulates axiopodium formation

The epididymal IS is a region characterized by a high level of ERK1/2 phosphorylation (pERK1/2)^20^,^21^,^22^. c-Src was shown to mediate androgen-dependent pERK1/2 in an epididymal cell line^23^. We, therefore, investigated whether the decrease in BC axiopodia length observed in c-Src KO mice was associated with a change in pERK1/2 in this region. As shown in Fig. 4d the IS in c-Src KO mice has reduced pERK1/2 compared with their WT littermates. These results indicate that the decrease in the number of BCs with axiopodia in c-Src KO mice correlates with a decrease in pERK1/2.

**Figure 5:**
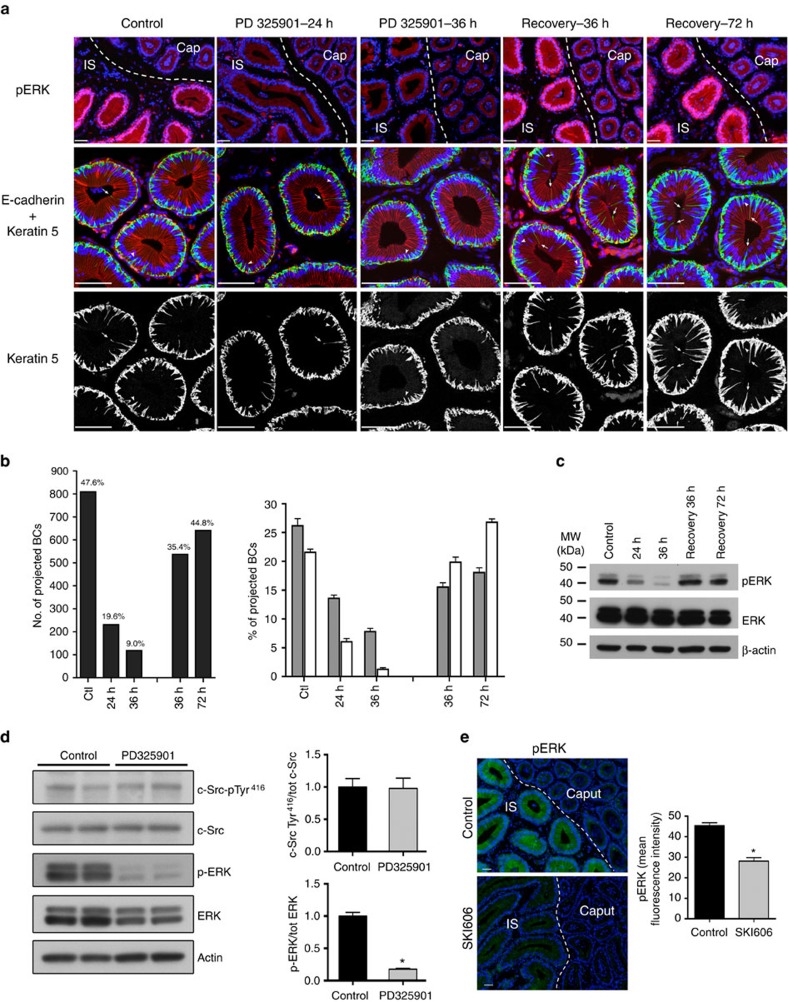
Role of MEK1/2–ERK1/2 in the formation of BC axiopodia. (**a**) The proximal epididymis was labelled for ERK1/2 (top panels), KRT5 (green: middle panels, white: bottom panels) and E-cadherin (red: middle panels), from control mice, mice treated with PD325901 for 24 and 36 h, and mice allowed to recover for 36 and 72 h. Arrows show long axiopodia that reach the apical pole of the epithelium (above the row formed by principal cell nuclei) and arrowheads show short axiopodia. (**b**) Left: quantification showing a progressive reduction in the number of BCs with axiopodia of all lengths after 24 and 36 h of PD325901 treatment and a progressive recovery after removal of the drug. Right: in control and PD325901-treated mice the number of BCs with long axiopodia is lower than the number of BCs with short axiopodia. During the recovery period, BCs with long axiopodia are more numerous than BCs with short axiopodia. (**c**) Western blot of proximal epididymis showing that PD325901 induced a progressive reduction in ERK1/2 phosphorylation, while total ERK1/2 remained unaffected. ERK1/2 phosphorylation returned back to normal after removal of the drug. β-actin was used as a loading control. (**d**) Left: western blots of epididymal IS showing that 36-h treatment with PD325901 had no effect on c-SrcTyr^416^ phosphorylation and total c-Src protein content. Here again a marked reduction in ERK1/2 phosphorylation was observed. Right: levels of phosphorylated c-Src and ERK1/2 were normalized to their respective total protein levels. Data are means±s.e.m. from four mice. *P<0.001 by ANOVA followed by Student's *t*-test. (**e**) Left: immunofluorescence detection of pERK1/2 showing a significant reduction in phosphorylation levels in the IS of mice after 36 h treatment with SKI606 compared with controls. Right: significant reduction in pERK1/2-associated mean pixel intensity in the IS of SKI606-treated mice compared with controls. *P<0.001. Scale bars, 50 μm.

We further analysed the role of pERK1/2 in the maintenance of BC axiopodia by treating WT mice with the MEK1/2 inhibitor PD325901 via daily intraperitoneal injections (10 mg kg^−1^). Immunofluorescence shows stronger pERK1/2 labelling (red) in the IS compared with the caput region of control mice (Fig. 5a, top panel, Control). A progressive reduction in pERK1/2 labelling is observed after 24 h of treatment with PD325901, and an almost complete disappearance of pERK1/2 is observed after 36 h in the IS. The progressive decrease in pERK1/2 by PD325901 is confirmed with western blot analysis, while total ERK remained unchanged (Fig. 5c). After a 36-h treatment with PD325901, mice were allowed to recover without the drug for 36 and 72 h. During that recovery period, pERK returned back to normal level (Fig. 5a, top panels, and Fig. 5c). BC morphology was assessed in epididymis sections double-labelled for KRT5 (green) and the lateral membrane marker E-cadherin (red; Fig. 5a, middle panels). The KRT5 labelling alone is displayed in black and white in the bottom panels of Fig. 5a. Quantification of several hundreds of BCs shows a progressive reduction in the number of BCs with axiopodia after 24 and 36 h, and a progressive increase in the number of BCs with axiopodia during the recovery period (Fig. 5b, left panel). Interestingly, the ratio of BCs with long axiopodia (Fig. 5b, right panel, white bars) to BCs with short axiopodia (Fig. 5b, right panel, grey bars) was reversed during the recovery period compared with controls. These results confirm the role of the MEK1/2/ERK1/2 pathway in the regulation of BC morphological plasticity.

The reduction in pERK1/2 that we observed in c-Src KO mice indicates that c-Src-related events are located upstream of MEK1/2. This is confirmed by the absence of effect of PD325901 on c-Src Tyr^416^ phosphorylation (Fig. 5d) while pERK1/2 levels are markedly reduced, and by a significant reduction in ERK1/2 phosphorylation, observed by immunofluorescence, in the IS of SKI606-treated mice compared with controls (Fig. 5e).

### Proposed model of BC axiopodium motility

**Figure 6:**
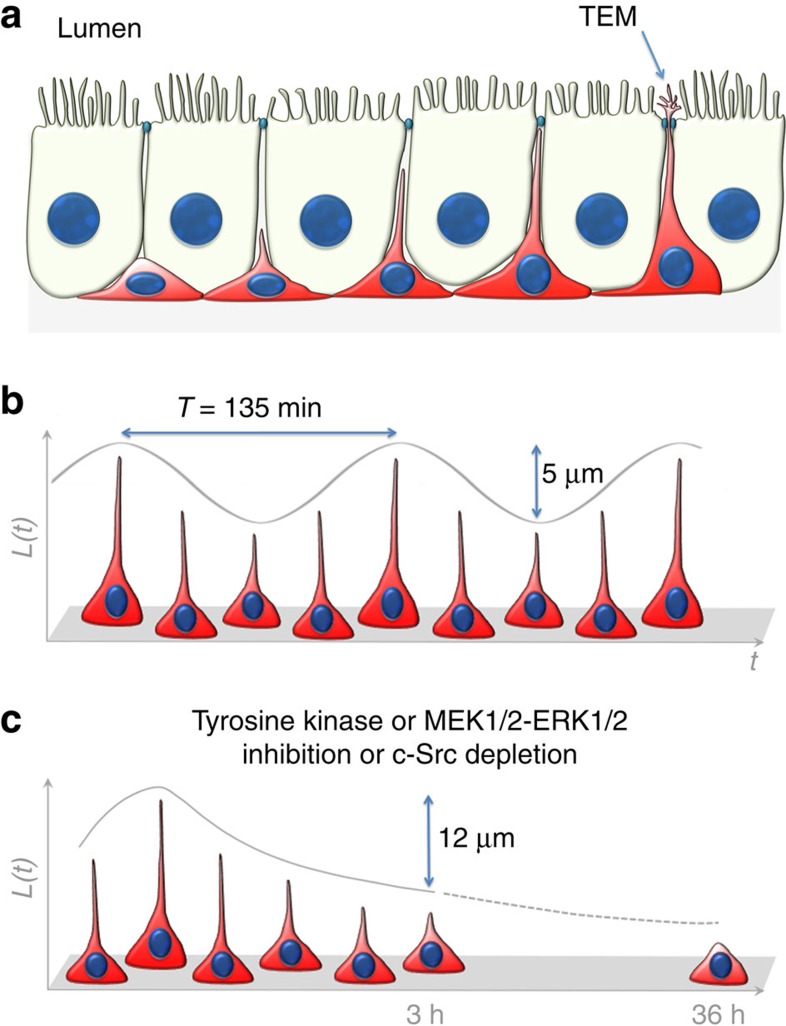
Model of BC plasticity. (**a**) BCs (red) with axiopodia of different lengths are present in pseudostratified epithelia. Some axiopodia can cross the tight junction barrier and then exhibit small TEM (arrow). (**b**) Variation of the length of individual BC axiopodia as a function of time under control conditions. Each axiopodium extends and retracts in a periodic pattern. On average, the period of oscillation is 135 min and the amplitude is 5 μm (see also immunofluorescence examination of the proximal epididymis (IS; Fig. 1b,c and Supplementary Movie 7). (**c**) Therapeutic tyrosine kinase inhibition, pharmacologic MEK1/2 inhibition or genetic depletion of c-Src induce a progressive retraction of the BC axiopodia. After 3 h, the axiopodia are shorter by an average value of 12 μm, and, after 36 h, most BCs have retracted their axiopodia.

Figure 6 shows our proposed model for the periodic pattern of elongation/retraction of BC axiopodia. BCs with several morphological characteristics can be visualized among adjacent epithelial cells in the proximal epididymis, from BCs that have no axiopodia to BCs with variable axiopodium lengths (Fig. 6a). Some BC axiopodia can cross the tight junction barrier and then exhibit small TEM (arrow) that are in contact with the luminal compartment. Figure 6b illustrates our 4D model of the structural plasticity of a given BC as a function of time. According to this model, each BC elongates and retracts its axiopodium in an oscillatory manner, a process that we name PAM (see also immunofluorescence examination of the proximal epididymis (IS; Fig. 1b,c and Supplementary Movie 7). The curve fit analysis shown in Fig. 3e estimates an averaged period for PAM of 135 min and amplitude of 5 μm, and a maximum speed of extension of 4.2 μm h^−1^. Figure 6c shows the retraction of BC axiopodia after therapeutic inhibition of tyrosine kinases with SKI606, pharmacologic inhibition of MEK1/2 or after c-Src depletion in KO mice.

## Discussion

Using a novel KRT5^Cre^/tdTomato^LoxP^ transgenic mouse that expresses tdTomato in BCs, we show that pseudostratified epithelia, including the trachea, epididymis, prostate and seminal vesicles, contain BCs that extend a narrow intercellular projection along their basal-to-apical axis in the direction of the lumen. These projections, which we term axiopodia, can cross the epithelial tight junctions to reach the lumen of the organ. In the epididymis, they then produce small TEM that are in contact with the luminal compartment. A 4D FIVM analysis of the structural plasticity of tdTomato-positive BCs in the epididymis revealed that each axiopodium constitutively retracts and elongates. The retraction/elongation movement of BC axiopodia follows an oscillatory pattern, a process that we call PAM.

The proximal epididymis receives lumicrine factors that are secreted by the testis and which, together with androgens, contribute to activating the MEK1/2-ER1/2 pathway^20^,^21^,^22^. In addition, the tyrosine kinase c-Src was shown to mediate androgen-dependent pERK1/2 in an epididymal cell line^23^. c-Src plays essential roles in sperm activation and in the regulation of epithelial cell differentiation in the epididymis^14^,^24^,^25^. Importantly, tyrosine kinase inhibitors, including SKI606, are currently used for the treatment of some cancers^18^. However, these drugs have adverse effects on male fertility^12^. We show here that tyrosine kinase inhibition with SKI606 induced axiopodia retraction in BCs. Our study, thus, provides a potential mechanism by which male fertility might be impaired following treatment with anticancer drugs that target the tyrosine kinase pathway. After a short treatment of 3 h, BC axiopodia were on average 12 μm shorter (approximately 30% shorter than the axiopodium maximal length); however, we anticipate that the amplitude of retraction would have become even greater with time if we could have visualized the cells for longer periods of time in the living animal. In agreement with this notion, we found using fixed tissues that most BCs had completely retracted their axiopodia after 24 and 36 h of treatment with the MEK1/2 inhibitor PD325901.

We recently showed that luminal factors secreted by the testis regulate BC intercellular projections in the mouse proximal epididymis^2^. As mentioned above, these factors activate MEK1/2 and ERK1/2 (refs 20, 21, 22), which we show here are the key regulators of PAM in BCs. In addition, c-Src might be one of the tyrosine kinases that participate in the impairment of male fertility resulting from cancer treatment. Indeed, our study shows that in the epididymis c-Src regulates BC axiopodia formation via the MEK1/2 pathway, in agreement with a previous study showing its participation in androgen-dependent pERK1/2 in epididymal cells^23^. Our results are also consistent with our previous study showing that c-Src KO mice have a poorly differentiated distal epididymis^14^.

We show here that BCs have the remarkable ability of changing their shape by first elongating a long and slender intercellular body projection (an axiopodium), and then by producing smaller TEM extensions. This dynamic plasticity implies a complex interplay between membrane protein and lipid composition, physical forces applied to the membrane surface, intracellular signalling pathways and cytoskeletal rearrangement, some of which have been implicated in the plasticity of other structurally dynamic cell types^26^,^27^. The formation of membrane protrusions is generally regulated by the actin cytoskeleton^26^; however, in some cells it is modulated by microtubules^28^. To determine whether BCs use actin filaments and/or microtubules to elongate their axiopodia and form apical microvilli will require additional work. In our study, the role of c-Src in modulating the formation of axiopodia in BCs is in agreement with a previous report showing abundant c-Src expression in platelets^27^, and the role of c-Src and ERK in the regulation of lamellipodium formation in migrating cells^26^. Activated c-Src is attached to the inner face of the plasma membrane and co-purifies with lipid rafts^27^. c-Src is an effector and regulator of specific receptors, including epidermal growth factor receptor (EGFR)^29^,^30^,^31^, and EGFR regulates the formation of lamellipodia^26^. EGF is among the luminal factors secreted by the testis^32^, and BCs express EGFR^33^. Altogether, these previous results and the present study indicate the potential participation of EGFR and lipid rafts via c-Src signalling in the membrane curvature that takes place at the site of membrane extension in BCs.

Membrane protrusions are often associated with cell migration. However, the BC main body remains anchored within the epithelium even after axiopodium formation and elongation. The unexpected highly dynamic property of BC axiopodia might ensure regular and efficient sampling and decoding of luminal signals. Because they are not always in contact with the luminal compartment, BCs are less likely to have desensitized receptors, therefore providing a uniform response to constant luminal signals. For example, in the rat epididymis BCs sense luminal angiotensin II via the angiotensin II type II receptor, which then triggers the production of nitric oxide, followed by activation the cGMP pathway and proton secretion in adjacent proton-secreting clear cells via a crosstalk mechanism^1^. Spermatozoa are a source of ANGII, and this complex intercellular communication network might provide a means by which clear cells maintain an acidic luminal environment that is essential for proper sperm maturation and storage. Interestingly, at a given time not all BCs are in contact with the lumen. However, they form an elaborate network at the base of the epithelium and are connected via gap junctions^3^,^4^,^6^,^34^,^35^. Thus, a few luminal-sampling BCs might serve as sentinels that could propagate signals to adjacent BCs, which would then regulate other cell types within the epithelium via crosstalk. This complex intercellular network would provide several control levels for the precise regulation of epithelial function by luminal signals.

In summary, we show here a previously unrecognized dynamic structural plasticity of epithelial BCs. In all pseudostratified epithelia examined, including the trachea, epididymis, prostate and seminal vesicles, BCs exhibit a narrow body projection, which we term an axiopodium, between adjacent epithelial cells in the direction of the lumen. Using the epididymis as a model system, we show that BCs constitutively extend and retract their axiopodium in an oscillatory manner, a process that we have named PAM. At a given time point, some BCs have their axiopodium in contact with the luminal side of the epithelium, and their ‘apical' surface is then characterized by the presence of small TEM. Pharmacologic or genetic inhibition of the s-Src and MEK1/2–ERK1/2 pathways and therapeutic inhibition of tyrosine kinases induced a progressive retraction of BC axiopodia. These previously unknown effects may explain observed toxicities of some tyrosine kinase inhibitors, and might also provide novel therapeutic opportunities. Furthermore, the unexpected highly dynamic motility of BCs might be part of a sophisticated communication network, allowing efficient sampling and decoding of luminal signals in pseudostratified epithelia.

## Methods

### Animals

KRT5^Cre^/tdTomato^LoxP^ transgenic mice were generated by mating FVB.Cg-Tg(KRT1-5-cre)5132Jlj/Mmnc mice (KRT5^Cre^, Mutant Mouse Regional Resource Centers) with the Cre reporter mice B6.Cg-Gt(ROSA)26Sor^tm14(CAG-tdTomato)/Hze^/J (tdTomato^LoxP^, Jackson Immunoresearch Laboratories). These mice express the red fluorescent protein, tdTomato, under the control of the bovine KRT5 promoter. Only F1 generations of mice between the ages of 90 and 120 days were examined in this study. WT (FVB/N) mice were used as controls. All animals were housed and used in compliance with the Institutional Animal Care and Use Committees of the Massachusetts General Hospital (MGH), and in accordance with the National Institutes of Health *Guide for the Care and Use of Laboratory Animals.* Animals had free access to water and were kept on a standard food diet *ad libitum*. Age-matched (49–56 days old) c-Src KO and their littermates were also examined and have been described previously^14^,^16^. Nembutal (Pentobarbital; 60 mg kg^−1^ wt ip) was used to anaesthetize the animals during experimental procedures.

### PCR genotyping

Tail biopsy specimens were collected from WTs, KRT5^Cre^, tdTomato^LoxP^ and KRT5^Cre^/tdTomato^LoxP^ animals. Genomic DNA was isolated using DNeasy Blood & Tissue kit from Qiagen (Valencia, CA, USA) following the manufacturer's protocol. The *Cre* gene was amplified using the following primers:

5′-CATTACCGGTCGATGCAACGAGTGATGAG-3′ (forward) and 5′-GAGTGAACGAACCTGGTCGAAATCAGTGCG-3′ (reverse) producing an amplicon of 408 bp. The *tdTomato* gene was amplified using the following primers: 5′-CTGTTCCTGTACGGCATGG-3′ (forward) and 5′-GGCATTAAAGCAGCGTATCC-3′ (reverse) producing an amplicon of 196 bp. PCR amplification was performed in 20 μl aliquots containing (in mM): 1.25 MgCl_2_, 1 × PCR buffer, 0.2 deoxynucleotide mix, 0.005 forward primer, 0.005 reverse primer and 1.25 U of Taq polymerase (all from Applied Biosciences). PCR amplification was performed with the following parameters: 8 min at 95 °C, 30 s at 95 °C, 30 s at 60 °C, 1 min at 72 °C for 35 cycles, followed by a 10-min final extension at 72 °C. PCR products were separated with gel electrophoresis on a 2% agarose gel containing Gelstar (Lonza, Rockland, ME).

### Tissue collection

Epididymis tissues were fixed by immersion in a paraformaldehyde–lysine–periodate solution containing 4% v/v paraformaldehyde, 75 mM lysine, 10 mM sodium periodate and 5% w/v sucrose in 100 mM phosphate buffered saline (PBS) at room temperature for 4 h, or overnight at 4 °C. Tissues were washed with PBS three times and stored in 0.02% sodium azide–PBS. Cryosections of 5 and 16 μm thickness were collected from epididymides that were cryoprotected in 30% sucrose overnight at 4 °C and embedded in Tissue-Tek optimum cutting temperature (OCT) compound (Sakura Finetek, Torrance, CA). Cryosections were cut using a Leica CM3050-S microtome (Leica Microsystems, Buffalo, IL) and collected onto Fisherbrand Superfrost Plus microscope slides (Fisher Scientific, Pittsburg, PA).

### Immunofluorescence

Single- and double-immunofluorescence labelling was preformed as previously described^1^,^36^,^37^. Primary antibodies used are against the following: KRT5 (Abcam, ab53121, 1:500 dilution), E-cadherin (BD Biosciences, 610181, 1:500 dilution), ZO-1 (a generous gift from Dr Eveline Schneeberger, 1:1), phospho-specific (Thr^202^/Tyr^204^) p44/42 MAPK (ERK1/2, Cell Signaling Technology, Cat# 4370, 1:100), total ERK1/2 (Cell Signaling Technology, Cat# 4695, 1:100), total c-Src (Cell Signaling Technology, Cat# 2109, 1:100) and phospho-specific (pTyr^416^) c-Src (EMD Millipore, PK1109, 1:50). A heat antigen-retrieval procedure was employed for phospho-specific (pTyr^416^) c-Src immunolabelling that consisted of microwaving the cryosections for 4 min in 10 mM TRIS and 1 mM EDTA at pH 9.0. Affinity-purified donkey FITC-conjugated anti-rabbit secondary IgG, donkey FITC-conjugated anti-rat secondary IgG and donkey Cy3-conjugated anti-mouse secondary IgG were used following the manufacturer's protocol (Jackson Immunoresearch Laboratories). Nuclei were labelled with 4,6-diamidino-2-phenylindole. Images were taken on a Nikon A1R confocal microscope equipped with a motorized piezo z stage. Multiple optical sections taken along the *z* axis were acquired to produce the extended focus images and 3D reconstructions shown in some figures. BC axiopodia were divided into four classifications. ‘Apical reaching' axiopodia were defined as projections that reach the lumen or the apical border of the epithelium. ‘Past nucleus' axiopodia were defined as projections that past the row formed by the nucleus of adjacent principal cells. ‘Short projections' were defined as axiopodia that are present on BCs but do not extend past adjacent principal cell nuclei.

### Pre-embedding electron microscopy labelling

Epididymides were collected from KRT5^Cre^/tdTomato^LoxP^ transgenic mice and were immersion-fixed in paraformaldehyde–lysine–periodate for 4 h. The tissues were washed in 1 × PBS, and the IS and caput were isolated. They were cryoprotected in 1 × PBS/30% Sucrose/0.02% Sodium Azide, OCT-embedded and sectioned at a 100-μm thickness. The sections were collected into 1 × PBS/0.02% Sodium Azide as free-floating sections. The sections were then washed to remove OCT and cryoprotectant in preparation for pre-embedding labelling.

Sections were first quenched with 0.3% H_2_O_2_ for 10 min to remove any endogenous peroxidase activity. They were then washed and blocked in a permeabilization buffer containing 1 × PBS/1% bovine serum albumin/0.2% Gelatin/0.05% Saponin for 1 h at room temperature. An anti-DsRed/tdTomato antibody (Clontech #632496), diluted at 1:50, was applied to the sections for incubation overnight at 4 °C. The sections were then washed with the permeabilization buffer, and incubated with an anti-rabbit biotinylated secondary antibody (Jackson Immunoresearch Laboratories, West Grove, PA, #711-065-152), diluted at 1:200, for 2 h at room temperature. Sections were washed with the permeabilization buffer and then incubated with a streptavidin/horseradish peroxidase solution (Vector Labs Vectastain ABC kit #PK-4001) for 2 h at room temperature. Sections were then post-fixed in 1 × PBS/1% glutaraldehyde/5% sucrose for 30 min and washed in 0.05 M Tris pH 7.6/7.5% sucrose in preparation for 3,3′-diaminobenzidine tetrahydrochloride (DAB-HCL, EMS #13082) reaction. Each section was transferred onto slides and was incubated in 1 ml of DAB-HCL solution for 10 min. One microlitre of a 1% H_2_O_2_ solution was added to each section to catalyse the reaction, and sections were monitored for reaction intensity using a stereomicroscope (Nikon SMZ645). When sufficient intensity was reached (usually in less than 3 min), the DAB-HCL solution was removed and the sections were washed with 0.05 M Tris pH 7.6/7.5% Sucrose to halt the reaction. A final series of washes in 0.05 M Tris pH 7.6 was then performed to prepare the sections for embedding.

DAB-HCL-treated sections were washed in 0.1 M sodium cacodylate and then incubated with 1% osmium tetroxide/0.1 M sodium cacodylate. The sections were then put through a series of dehydration washes in ethanol and infiltrated with eponate resin. Eponate-infiltrated sections were placed flat on slides in drops of eponate, cover-slipped and baked at 60 °C overnight. The resulting eponate films containing the sections were then removed from the coverslip and slide. Regions containing the IS were then isolated and cut out of the film and placed flat in column molds in eponate and baked again overnight at 60 °C.

Sections embedded in column molds were sectioned on a Leica ultramicrotome. Ultrathin (98 nm) sections were serially collected onto oval slot grids (two to three individual sections per grid) and imaged using a JEM-1011 transmission electron microscope (JEOL, Tokyo, Japan). Each images was then rotated and aligned using the Fiji build of ImageJ and exported as individual tiff files. The resulting grey-scale tiff files were inverted and cropped using Photoshop, and a 3D reconstruction was rendered for each BCs using the Volocity software.

### Intravital microscopy

KRT5^Cre^/tdTomato^LoxP^ mice were used in combination with two-photon FIVM^8^,^9^,^10^,^11^ to visualize BC in real time *in vivo*. FVIM provides significant advantages over single-photon confocal microscopy, including reduced phototoxicity and photobleaching, as well as high lateral and axial resolution in combination with large penetration depth. In addition, the epididymis provides unique advantages for the study of intact tissues *in vivo* because it is located in the scrotal sac, which can easily be externalized and is not subjected to motion artefacts caused by heartbeat and respiration. KRT5^Cre^/tdTomato^LoxP^ mice underwent a minimal surgical procedure to exteriorize one epididymis. A tail vein cannulation was set up to deliver vehicle, the tyrosine kinase inhibitor, SKI606 (also known as Bosutinib; 30 mg kg^−1^), or additional pentobarbital if needed. Images of BCs from the proximal epididymis (IS) were acquired on a customized Olympus FV1000MPE multiphoton laser scanning confocal microscope using a × 25 (numerical aperture=1.05) water immersion objective. TdTomato was excited with a tunable Ti:sapphire laser at 765 nm, and a FV10-MRV/G filter was used to collect emitted light at 520±20 nm. A z-stack (z-step=1 μm) of 512 × 512 images (0.33 μm per pixel) was acquired every 15 min. The length of each BC was measured at each time point from six control and three SKI606-treated mice using the Volocity (v. 6.3) software. After FIVM imaging, both epididymides were collected and fixed by immersion, as described above.

### PD325901 administration

Adult male WT mice were divided into a control group (*n*=10) and a PD 325901-treated group (*n*=10). For the PD-treated group, PD325901 was given by intraperitoneal (i.p.) injection with a dosage of 10 mg kg^−1^ per day every 12 h for 12, 24 and 36 h (ref. 38). One epididymis was harvested and fixed by immersion, and the other epididymis was processed for protein and mRNA extractions. In a separate ‘recovery' cohort, mice were treated with PD 325901 for 36 h, one epididymis was removed and fixed by immersion and the mice were allowed to recover without further treatment for an additional 36 and 72 h. The other epididymis was then harvested and fixed by immersion. Western blot and PCR samples were collected in a separate group of mice.

### Statistical analysis

Data were analysed by ANOVA followed by Student's *t*-test for unpaired values. A value of *P*<0.05 was considered significant. Data were expressed as the means±s.e.

## Additional information

**How to cite this article:** Roy, J. *et al*. Tyrosine kinase-mediated axial motility of basal cells revealed by intravital imaging. *Nat. Commun.* 7:10666 doi: 10.1038/ncomms10666 (2016).

## Supplementary Material

Supplementary Information Supplementary Figures 1-2

Supplementary Movie 1 Epididymis from a KRT5Cre/tdTomatoLoxP mouse labeled for KRT5. 3D reconstruction from a stack of 0.1-µm interval images, acquired using a Nikon A1R laser scanning confocal microscope. KRT5 (green) labeling is present in all tdTomato-positive cells identifying them as BCs. Several BCs with long axipodia are seen.

Supplementary Movie 2 BC axiopodia can cross the tight junction barrier. 3D reconstruction of a tdTomato-positive BC (red) by laser scanning confocal microscopy of a 16-µm cryosection of the proximal epididymis. Tight-junctions were labeled in green with ZO-1. The BC axiopodium is in contact with the luminal side of the epithelium. Nuclei are labeled in blue with DAPI.

Supplementary Movie 3 BC axiopodia can cross the tight junction barrier. 3D reconstruction of a tdTomato-positive BC, superimposed on a differential interference contrast (DIC) image, showing that its axiopodium is in contact with the luminal compartment. Nuclei are labeled in blue with DAPI.

Supplementary Movie 4 3D electron microscopy visualization of the apical region of a BC. Epididymis was immunostained using an antibody that recognizes tdTomato and a stack of ultrathin serial sections were obtained by electron microscopy. Images from these serial sections were superimposed using Fiji and imported into Volocity software to generate a “3D” projection view of the cells. The “apical” pole of this BC is in contact with the luminal compartment and shows the presence of numerous short transepithelial microvilli. Longer stereocilia emanating from adjacent principal cells are also seen.

Supplementary Movie 5 Mouse proximal epididymal tubule visualized by 2-photon fluorescence intravital microscopy (FVIM). A stack of optical images was acquired along the Z-axis through the entire thickness of a portion of the tubule.

Supplementary Movie 6 Single BC visualized in the live animal by 2-photon fluorescence intravital microscopy (FVIM). 3D reconstruction of a single BC from a high-magnification Z-stack series showing its axiopodium that extends in the direction of the lumen.

Supplementary Movie 7 3D Model of BC plasticity. Animation of the BC shown in Figure 2c and Movie 2 illustrating the periodic axial motility (PAM) of its axiopodium. Each axiopodium extends and retracts in an oscillatory manner.
