# Supplementary material for: Tyrosine kinase-mediated axial motility of basal cells revealed by intravital imaging
Source: Nat Commun. 2016 Feb 12;7:10666. doi: 10.1038/ncomms10666 (PMC4754344; doi:10.1038/ncomms10666)
Supplement: Supplementary Information — Supplementary Figures 1-2 [file ncomms10666-s1.pdf]

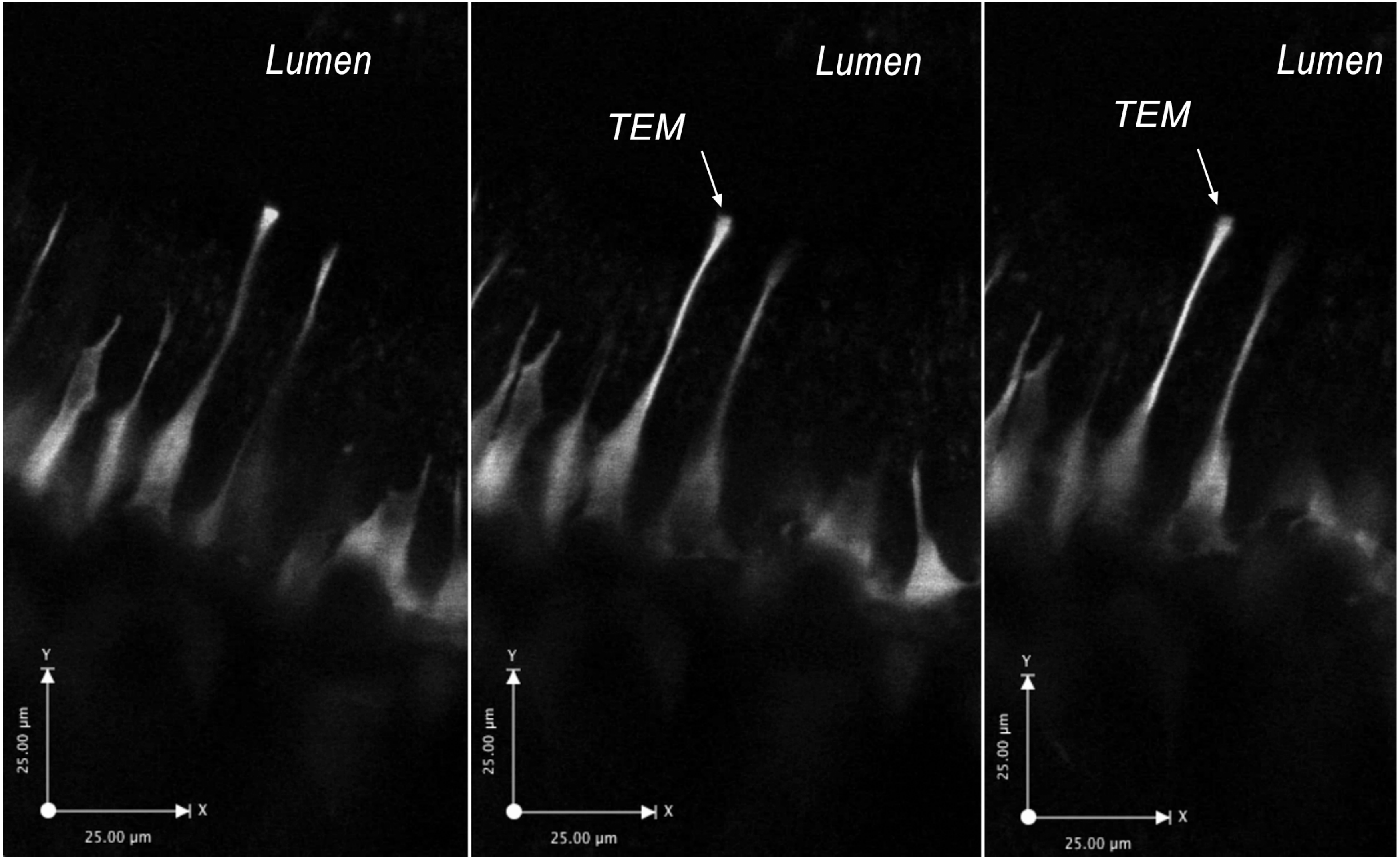

Supplementary Figure 1) Mouse epididymis visualized by 2-photon fluorescence intravital microscopy (FVIM). Three optical images were acquired at the level of the lumen along the Z-axis at .5 µm intervals, using a customized Olympus FV1000MPE multiphoton laser scanning confocal microscope. Several tdTomato-positive BCs are seen with their axiopodia that extend toward the lumen. The arrows in the middle and right panels indicate small projections located at the tip of the BC axiopodium.

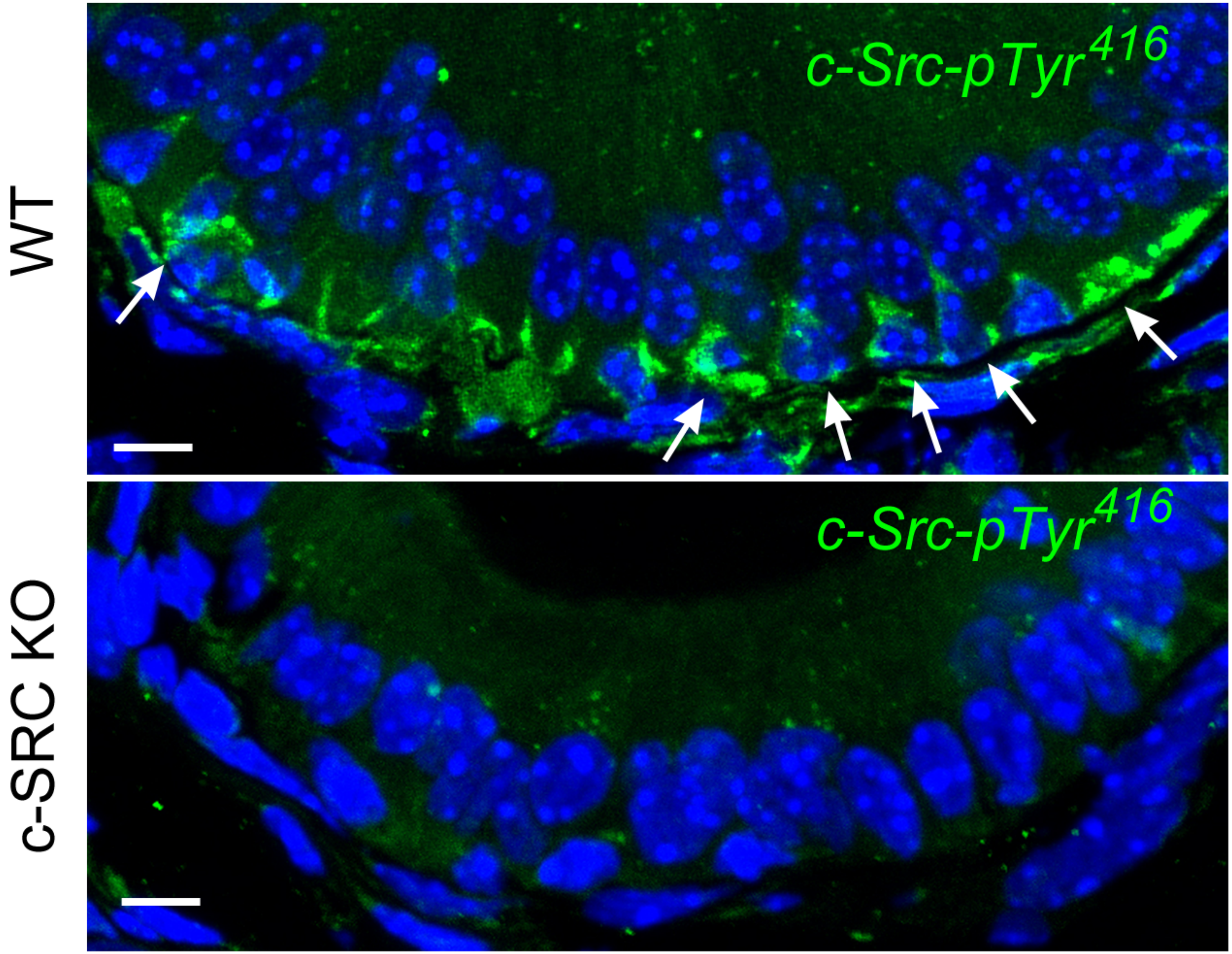

Supplementary Figure 2) Immunofluorescence labeling using an antibody that recognizes c-Src when phosphorylated at the Tyr416 residue in the IS of WT and c-Src KO mice. In WT mice, several basal cells are positive for c-Src-pTyr^{416} (arrows). In contrast, no labeling is seen in the IS of c-Src KO mice confirming specificity of the antibody. Scale bars = 5 µm.
